# What causes non-adherence among some individuals on long term antiretroviral therapy? Experiences of individuals with poor viral suppression in Uganda

**DOI:** 10.1186/s12981-018-0214-y

**Published:** 2019-01-21

**Authors:** Dominic Bukenya, Billy Nsubuga Mayanja, Sarah Nakamanya, Richard Muhumuza, Janet Seeley

**Affiliations:** 1 MRC/UVRI and LSHTM Uganda Research Unit, P.O. Box 49, Entebbe, Uganda; 2 London School of Hygiene and Tropical Medicine, Keppel Street, London, WC1E 7HT UK

**Keywords:** Antiretroviral therapy, Adherence, Long term ART, Uganda, In-depth interviews

## Abstract

**Background:**

Antiretroviral therapy (ART) use by people living with HIV reduces HIV transmission, morbidity, mortality, and improves quality of life. Good ART adherence is required to achieve these benefits. We investigated how the environmental, social, economic and behavioural experiences of people living with HIV with poor viral suppression could explain their non-adherence to long term ART.

**Methods:**

This qualitative cross-sectional study was conducted in Uganda between September 2015 and April 2016. Thirty individuals on ART for 5 years or more (10 on first line and 20 on second line), with poor viral suppression, were randomly selected from a cohort of people living with HIV on ART. In-depth interviews about ART; awareness, adherence counselling, obstacles to daily adherence and regimen switches were conducted. Emerging themes from the interviews transcripts and field notes were identified and thematic content analysis done. Participants’ consent, compensation, confidentiality and study ethical approvals were ensured.

**Results:**

We found that poor adherence to long term ART was due to: travel for work or social activities, stigma, receiving little or no continuous ART adherence education, alcohol consumption and use of alternative ‘HIV cure’ medicines. Other reasons included; ART side effects, treatment fatigue, belief that long-term ART or God can ‘cure HIV’, and food security.

**Conclusions:**

Achieving optimal ART benefits requires continuous provision of ART adherence education to individuals on long term ART. This helps them overcome the challenges related to living with HIV: worries of food insecurity, alcohol misuse, economic hardship, and beliefs in HIV cures and use of unproven alternative HIV treatments. People living with HIV who travel require adherence support and larger quantities of ART refills to cover their time away.

## Introduction

Antiretroviral therapy (ART) use in HIV management has reduced morbidity and mortality among people living with HIV. ART also improves life expectancy and quality of life for people living with HIV, while the resultant viral suppression reduces the HIV transmission risk [1–5]. Good adherence, defined as following the recommendations made by the treatment provider on timing, dosage and frequency of medication taking [6], is a prerequisite to realising these ART benefits [7–12]. However, adherence remains a challenge for many people living with HIV [1, 13–15].

A systematic review of individual and contextual factors affecting retention and adherence among pregnant and post-partum women living with HIV found that religious beliefs and use of alternative medicines discouraged ART adherence [16]. Similar findings have been reported from other studies involving different populations [17–19]. Other systematic reviews of barriers to ART adherence in developing and developed countries showed that the fear to disclose HIV status, treatment suspicion together with the desire to avoid taking medication in public places among others, negatively affected adherence [20, 21]. In Botswana, 31% of the patients on ART found it hard to cope with a long duration on ART due to: financial difficulties, forgetfulness, travel, side effects and being food insecure [13]. Similar findings have also been reported in Democratic Republic of Congo, Zambia, Uganda, Kenya and Tanzania [22–27]. There are contrasting reports on the association between duration on ART and adherence. Whereas some studies have reported decreasing adherence with increasing duration on ART [2, 14, 17, 21, 28–31], others have reported an increase in adherence as people stay longer on ART [29, 32–36].

Despite the available evidence, few studies have examined the underlying environmental, social, economic and behavioural circumstances among people living with HIV with poor viral suppression that could explain their non-adherence to ART. In addition, to achieve the UNAIDS 2020 targets (90% of all people living with HIV will know their HIV status, 90% of those people will be on ART and 90% of them will be virally suppressed [37]), issues of living with HIV and long term ART use to ensure viral suppression need to be explored further [38]. In this paper we examine the experiences of people living with HIV with poor viral suppression and how different circumstances could explain their non-adherence to long term ART.

## Methods

### Study design and settings

This cross sectional qualitative study was nested in the Complications of Long-term Antiretroviral Therapy (CoLTART) cohort study that was conducted in Kalungu and Wakiso districts in Uganda. The CoLTART cohort was a 3 year cohort established in 2013 to study the long-term clinical and virological consequences of ART and HIV among African patients [39]. In Kalungu district, the CoLTART participants were recruited from the former Rural Clinical Cohort (RCC) in Kyamulibwa, where ART was introduced in 2004 [40]. In Wakiso district, the CoLTART participants were recruited from the Entebbe site of the former Development of Antiretroviral Therapy in Africa (DART) Trial, where ART has been provided since 2003 [41]. The CoLTART cohort study ART clinics were well equipped and staffed. In addition, HIV care medications including ART and cotrimoxazole were always in stock and people living with HIV were offered ART adherence health education. During the pre-ART initiation health education sessions, participants and their treatment supporters were prepared for ART initiation [42]. Continuous post ART initiation health education was offered to people living with HIV at every ART refill visit to encourage them to adopt safe sexual behaviours and adherence to ART. The CoLTART cohort therefore provided us with a platform to investigate the underlying environmental, social, economic and behavioural circumstances contributing to non-adherence to long term ART. Long term ART in this paper is defined as being on ART for 5 years and above.

### Study population and sampling

At enrolment into CoLTART, 110 (12.5%) of the participants had viral loads ≥ 1000 copies/ml, 74 (67.3%) were on first line ART and 36 (32.7%) on second line ART [43]. A random stratified sampling technique was used to select 30 participants who had been on ART for 5 or more years. We aimed for more participants (20) on second line ART than on first line ART (10) due to their limited treatment options in Uganda where third line ART is not readily available. All the selected participants’ plasma viral load was greater or equal to 1000 copies/ml and were evenly distributed between the two CoLTART cohort study sites.

### Data collection and management

In-depth interviews were used to collect qualitative data from September 2015 to April 2016. A male and a female, experienced, local interviewers conducted the interviews which were audio recorded, but the interviewers also wrote field notes. The interviews lasted between an hour to an hour and a half and were conducted in Luganda, a widely spoken and understood language in the study area. The interview guide included topics about ART awareness, pre-ART and post-ART initiation adherence counselling, obstacles to daily ART adherence and circumstances leading to switch of regimen for those on second line ART.

Interviewers developed detailed interview accounts in English of each interview using both the audio recordings and the field notes soon after collection. Interview accounts were prioritised over interview transcripts because they provided additional information about the circumstances of data collection beyond transcribed recordings. The two interviewers were experienced in this approach and wrote detailed interview accounts which they shared to check clarity and completeness to ensure minimal errors and consistency. Thereafter, the study co-principal Investigator read each interview account and provided feedback to the interviewers while the principal investigator read a sample of these. The interviewers provided the additional information by listening to the audio recording again and/or re-reading through the field notes. The final interview accounts were kept on password protected computers during analysis and stored on a secure server, where access was restricted to the study team.

### Data analysis

The final interview accounts were read to identify emerging themes, which were discussed and a final coding framework was agreed on. The first coding level involved manual coding for circumstances leading to non-adherence to long term ART. The second level of coding was to identify the patterns of these long-term ART non-adherence circumstances and their main drivers. This coding level also involved extraction of illustrative quotes from the interviews presented in this paper.

### Ethics approval and consent to participate and for publication

The Uganda Virus Research Institute Research and Ethics Committee and the Uganda National Council for Science and Technology approved this study.

Prior to the interview, signed written informed consent was obtained. In case of non-literate participants, a witnessed thumb-printed informed consent was obtained. Consent was provided for participation and for publication, including giving consent to the following statement: ‘I agree that anonymised direct quotes from the ART adherence interview may be used in the public reporting of these interviews’. Study participants were compensated for their time and they were assured of confidentiality.

## Results

Of the 1095 individuals enrolled into the CoLTART cohort, 220 (20.1%) were on the second line ART, 110 (10.5%) had HIV RNA viral load of 1000 copies/ml or higher, including 36 on second line ART, as noted above. Between September 2015 and April 2016, in-depth interviews were held with 30 participants (10 on first line and 20 on second line ART); mean age 41.7 years (standard deviation 8.7), 18 (60%) females and about 80% were Christians. Almost half (47%) of the participants were either married or in a relationship, and a third were either separated or divorced. Half was educated up to primary level.

### Working away from home and work-related mobility

Sixteen of the thirty respondents reported that work and social related travel affected their adherence to ART. Some, especially men (7/12), explained that they often worked and spent long periods away from their homes and occasionally carried fewer ART tablets than they needed or feared the stigma of being seen taking daily medications as this would raise suspicion of one being HIV infected. Others missed refill appointments either because of work commitment or failure to raise transport fares or both. Three men reported that their ART drugs were finished while working in Juba, South Sudan. When they came back for refills, their ART regimen was switched after the health workers had rebuked them for failing to take ART. Only a few of the women (4/18), mainly urban, who worked in bars, shops or operated small restaurants reported being non-adherent to ART because they either worked far away from their homes or they finished the days’ work late and could not take their ART medication at their work places.

*“There are times when I swallow late particularly when I have many customers. I fear to swallow in the presence of other people”,* female aged 23 years, on first line ART.

Both men and women attributed the failure to take daily ART dose at the work place to being busy, fearful of anticipated stigma and or raising suspicion of HIV infection.

*“At times you get trips to travel, like travelling to Juba (South Sudan) for work and drugs get finished yet coming back is hard. This also contributes to my non*-*adherence to treatment”,* male aged 44 years, on second line ART.

*“Sometimes, I don’t have anything to eat especially in the morning. I attend high profile meetings and during these meetings we are not allowed to move out. During these meetings, they serve biscuits, yet I am diabetic I can’t take that”,* male aged 51 years, on first line ART.

*“There are times when I take the morning dose and fail to swallow the evening dose particularly when I’ve travelled far away from home. I am a taxi motorcyclist and get home late”,* male aged 33 years, on second line ART.

In addition, some female participants (5/18) who reported non-adherence to ART after several years on treatment, attributed their difficulties with taking their drugs to their travels to care for sick relatives and or attending funeral or wedding ceremonies. They explained that sometimes they would end up staying longer than the drugs they had carried and yet they could not raise the transport fares back to their ART clinics. Four women on second line ART regimen reported that they had moved away for a period of 1 or more months and during this time they did not take any ART medicines. When they returned, the health workers rebuked them for failing to take their drugs and ran tests to find out whether the ART was still effective and subsequently, two of them had their ART regimen switched.

*“I am involved in business and work in markets. I carry drugs for two months thinking I would be back then but I end up spending three months”*, female aged 28 years, on second line ART.

*“When I come to the shop without my drugs hoping to go back before the time for my drugs, sometimes I end up staying 3*–*4 extra hours. Yet the counsellor warned us during the pre*-*ART initiation adherence counselling not to take the tablets (ART) after delaying beyond 2* *h”,* female aged 40 years, on second line ART.

### Relaxed continuous ART adherence education/counselling

Even though continuous ART adherence education was to be offered at every ART refill visit, this was not always the case. About a third (9/30) of the participants reported missing taking their ART medications sometimes. They reported that when they came for ART refills, they were simply asked whether they were still adherent and how they were doing. They also explained that the continuous ART adherence education was only offered to those who were suspected to be non-adherent based on stagnant or decreasing CD4 cell counts.

*“They no longer offer adherence health education and I also relaxed in taking my medication (ART). I think they think we are now well versed”,* male aged 54 years, on first line ART.

*“They just ask if you are taking your drugs well and then they give you the drugs. They don’t repeat the ART adherence health education messages. I think they know that you understood clearly what to do but with ART you need to be motivated to continue taking it daily”,* female aged 28 years, on second line ART.

### Alcohol use or misuse

Most respondents (9/12) reported alcohol use, regardless of the ART regimen line they were on, however only two reported skipping taking their medication due to alcohol use. They argued that in the pre-ART and post-ART initiation adherence education sessions, they were asked to reduce alcohol use not to cease its use completely.

*“Sometimes, I take alcohol excessively and not only forget to take food but also drugs. The health care workers even switched my drugs to the type I am taking now. I still struggle to take the tablets”,* male aged 37 years, on second line ART.

In contrast, no female respondents reported using alcohol. The women reported that although they took alcohol prior to ART initiation, they stopped it completely when told to do so by the counsellors.

*“I stopped taking alcohol completely shortly before I was put (initiated) on these HIV drugs (ART). The counsellor told us that the drugs were incompatible with alcohol and that one had to choose between drugs and alcohol. That is when I chose drugs and gave up on alcohol. I have never tried it again”,* female aged 32 year old, on first line ART.

### Availability of other HIV treatment options

Eight respondents, mostly females (5/8) reported that their adherence to long term ART was sometimes interrupted because of the use of alternative medicine options. They explained that there were radio advertisements of alternative medicines which reportedly cure all illnesses and told them that taking too much western medication was dangerous to their health.

*“I am asthmatic and I sought herbal medicines for this at one time. When I reached they checked using the palms of their hands. After checking they told me that I had pressure too. I was given drugs/herbs in tea leaves form and they told me it cures all illnesses. During the time I took this herb, I ceased taking the tablets (ART)”,* female aged 53 years on second line ART.

### Treatment fatigue

Seven respondents reported that their non-adherence to ART was out of “*obutaffaayo”* (laziness) or simply being tired of taking ART endlessly. Such participants accorded themselves drug holidays. For some this behaviour contributed to becoming non-adherent.

*“I swallowed at the beginning but when I became stronger, I stopped taking. I could not swallow as I was supposed to”,* male aged 42 years, on second line ART.

It was apparent that for some study participants ‘becoming tired of ART’ was a proxy for other barriers to adherence, such as stigma or fears about side effects.

### Experiences or fear of ART side effects

Six respondents, two on first and four on the second line ART regimen, reported that experiences or fear of treatment side effects led to their non-adherence to ART. They explained that although during the pre-ART initiation health education, the health workers informed them that the side effects would be mild, some of them (3/6) had experienced very severe side effects. The severity of the ART side effects compelled these participants to discontinue ART to get relief. Among the reported severe side effects were: body deformities, severe headaches, nausea, numbness, diarrhoea and skin rashes.

*“The drugs used to give me problems, so they changed them. Even now, it still gives me black swollen patches on the skin, I vomit the food I have eaten. For this reason, sometimes I don’t take the medication”,* male aged 28 years, on second line ART.

*“I sometimes don’t take my medicine when I am leaving home. The medicine makes me feel bad and I fear collapsing in the public because I work in the market”,* female aged 37 years, on second line ART.

In addition, four respondents who had not experienced any ART side effects, sometimes also missed taking their ART and/or tuberculosis medication because they feared the possibility that side effects may occur. They reported that they had heard about the ART side effects from friends who had such experiences, the ART adherence education sessions from health workers and from radio programmes. Irrespective of ART regimen line, these participants’ fears resulted in three of them completely discontinuing ART.

### Belief that God or ART can cure HIV

Five of the 18 female respondents reported ceasing taking ART because they believed that either God or ART drugs had cured their HIV infection. Three female Pentecostal Christians reported that they had been cured by God, and therefore had no need to continue taking ART. They also added that their pastors asked them to stop taking ART drugs.

*“I stopped taking the drugs because I believed that God had cured my HIV infection”,* female aged 33 years, on first line ART.

Others said that they believed and felt cured of HIV infection after being on ART for years since they no longer had any illnesses and carried out their daily activities without any hindrances which was proof that the ART had cured the HIV infection.

*“I believe that when you adhere to the medication and you resist from all worldly doings, these drugs reach a time and they cure the HIV completely”,* female aged 44 years, on first line ART.

These two women alleged that they were never told the truth during ART adherence education sessions when they were told that ‘ART did not cure HIV infection’.

*“if you faithfully follow the doctor’s instructions about taking the tablets (ART), they (ART tablets) make the (HIV) virus so weak to the point of curing it completely”,* female aged 37 years on first line ART.

### Food insecurity

Four of the 15 urban respondents reported skipping taking their ART medicines because they did not have anything to eat prior to or soon after taking their medications. This was not mentioned by the rural respondents. The urban participants had become food insecure following job losses. They explained that sometimes they got food to prepare several hours past the time of taking the day’s ART dose yet during their ART adherence education sessions, they are cautioned against taking medications on an empty stomach. They added that every time they took ART drugs on an empty stomach, they experienced side effects. A combination of food insecurity leading to variations in meal time and fear and/or experience of drug side effects led to non-adherence to ART in the long run.

*“I take when I have food and when I have no food I stop taking the drugs”,* male aged 42 years, on second line ART.

*I sometimes lack food as a result I fail to take medication in fear of medicine affecting me badly”,* female aged 43 years, on first line ART.

### Incarceration

Three of the 12 male respondents reported that they frequently got imprisoned which affected their adherence to ART medications. They explained that while in prison, they sometimes missed taking their ART drugs. This was partly due to lack of food/water with which to take their drugs and being incarcerated at far away facilities from their ART refill clinics. They reported that prison authorities only served two meals a day: at mid-morning and early evening hours, times that were not compatible with their ART taking time and resulted in non-adherence to ART medications.

*“When you are in prison, they can’t give you water and food to take your medicine every day. I am often taken to prison because of a land wrangle with my neighbour. Whenever, I am in prison, I miss taking my drugs (ART)”,* male aged 44 years, on second line ART.

## Discussion

In this cross-sectional qualitative study among Ugandan adults on ART for 5 years or more but with poor viral suppression, majority of the respondents attributed their poor adherence to: working away from home, stigma and non-HIV serostatus disclosure, relaxed continuous ART adherence education/counselling and alcohol use or misuse. In addition, a few others attributed their poor adherence to availability of other HIV treatment options, treatment fatigue, experiences or fear of ART side effects, belief that God and ART can cure HIV, food insecurity and incarceration.

We found that as people’s health improved on ART, some individuals move to places far from their ART refill clinics in pursuit of work to earn an income resulting into missing ART refill appointments. The missed ART refill appointments could be due to either busy work schedules, lack of transport fares or could even have been used to mask either perceived or experienced stigma [44]. These challenges are likely to affect more people, as increasing numbers are diagnosed as living with HIV as universal treatment access is rolled out in the effort to reach the UNAIDS 2020 targets [45].

Resuming work could be an indicator of the normalisation process which is a shift from the diseased to healthy/normal self-identity. An ‘HIV-diseased identity’ is characterised by consistent routine drugs taking, dos and don’ts some of which could result into involuntary HIV serostatus disclosure. To avoid this and the resultant stigma either experienced or imagined, some patients could decide to find work at faraway places resulting in non-adherence to long term ART. Studies elsewhere found that longer duration on ART together with poor health care workers’ communication were significant predictors of non-adherence [24, 46–50]. The solution to this lies in consistent continuous health education and addressing the challenges of providing an ART delivery model where stable ART patients can get larger ART refill quantities or allowed to refill from any nearby ART delivering clinic.

A longer duration on ART can improve people’s health and also promote changes in perceived stigma [51]. Due to high self-stigma levels, individuals who worked away from their homes feared to be seen taking their pills at their work places by claiming to be very busy. Similar findings were reported from studies done in Botswana, Ethiopia, Kenya and Nigeria, but among individuals on ART for less than 5 years. In these studies, it was found that non-adherent ART patients attributed non-adherence to feeling healthy, being too busy to interrupt work schedules and stigma [52–55].

Providing continued ART adherence education remains an important challenge to long term ART adherence in developing countries faced with a high HIV burden. These countries have low staffing levels amidst an ever-increasing number of patients on ART. With the increasing number of patients on ART, health care workers need to prioritise the new patients recently initiated on ART, and this challenge will increase as more people living with HIV take ART [46, 56–58]. Not reinforcing messages or sharing new information about ART could affect adherence as suggested by some of our respondents, a finding corroborated by a study from Lesotho where people living with HIV who were stable on ART were receiving fewer adherence education sessions, contributing to non-adherence. However, these people living with HIV had been exposed to ART for less than 5 years [54].

The finding that alcohol misuse led to non-adherence to long term ART especially among men is consistent with studies from Uganda, Zambia, and Democratic Republic of Congo that reported that as health improved, people resumed alcohol use [29, 52, 54, 59]. A systematic review and meta-analysis of studies conducted in Ethiopia also reported that alcohol use was among the risk factors for ART non adherence [60].

The beliefs in available “alternative HIV treatment options” which we observed in our study was also shown in research in Ethiopia which reported that periodic religious rituals were barriers to ART adherence whether in the short, medium or long term perspective [61]. Traditional healers have also been reported to promote ART non-adherence although the majority of the participants in these studies had been on ART for durations shorter than 5 years [23, 25, 54, 62, 63].

We also found that fear and/or experience of ART side effects led to non-adherence to long term ART. Other studies have reported similar findings especially among food insecure patients [23, 48, 49, 54, 55, 62] though almost all these studies did not include people on ART for long durations who were experiencing poor viral suppression.

Beliefs that God and ART can cure HIV have been reported in an African and Asian cohorts comparative analysis [21]. Research in Ethiopia reported that some ART patients skipped their daily doses due to religious ritual observance, but also because of a belief that that God had cured them of HIV [18, 61, 64]. Contrary to these findings, a study in Uganda found that being religious facilitated long term ART adherence [58].

In our study, food insecurity was reported to contribute to non-adherence to long term ART only among the urban residents. Studies in other resource limited settings in the Democratic Republic of Congo, Namibia, Kenya’s capital city Nairobi and resource rich Atlanta in USA have also reported that food insecurity was common among the poorest people living with HIV and an important barrier to ART adherence [23, 28, 47, 48, 62], underlining the importance of livelihood enhancement and general development in supporting people living with HIV [49, 53].

## Conclusion

Although we did not aim to generalise our findings to other populations, our study provides insights relevant beyond our study population of individuals on long term ART with poor viral suppression because people living with HIV and on ART can easily move in and out of adherence/non-adherence categorisation. Also regardless of the adherence category one takes up, all people living with HIV and on ART are faced with daily demands of ensuring continued adherence. To achieve optimal benefits from ART, health workers should continue providing ART adherence education to individuals on long term ART who are vulnerable to challenges of the normalisation process like resuming work activities, stigma re-emergency and treatment fatigue among others. These challenges are bound to become more prevalent in view of the UNAIDS 2020 targets. Individuals who work far from their homes and ART clinics could have refills of larger quantities than usually dispensed or considered for temporary transfers arrangements to ART clinics near their places of work. In addition, embedding economic empowerment and managing alcohol abuse among individuals on long term ART would go a long way to ensure ART adherence. Efforts geared towards improving access to HIV diagnosis, linkage to care, ART initiation, retention and adherence call for more investment in the health sector performance monitoring.
